# Structural studies on MRG701 chromodomain reveal a novel dimerization interface of MRG proteins in green plants

**DOI:** 10.1007/s13238-016-0310-5

**Published:** 2016-09-08

**Authors:** Yanchao Liu, Hong Wu, Yu Yu, Ying Huang

**Affiliations:** 1 State Key Laboratory of Molecular Biology, National Center for Protein Science Shanghai, Shanghai Science Research Center, Shanghai Key Laboratory of Molecular Andrology, Institute of Biochemistry and Cell Biology, Shanghai Institutes for Biological Sciences, Chinese Academy of Sciences, Shanghai, 200031 China; 2 University of Chinese Academy of Sciences, Beijing, 100049 China; 3 State Key Laboratory of Genetic Engineering, Collaborative Innovation Center of Genetics and Development Institute of Plant Biology, School of Life Science, Fudan University, Shanghai, 200433 China

**Keywords:** MRG701, chromodomain, homodimer

## Abstract

MRG proteins are conserved during evolution in fungi, flies, mammals and plants, and they can exhibit diversified functions. The animal MRGs were found to form various complexes to activate gene expression. Plant MRG1/2 and MRG702 were reported to be involved in the regulation of flowering time via binding to H3K36me3-marked flowering genes. Herein, we determined the crystal structure of MRG701 chromodomain (MRG701^CD^). MRG701^CD^ forms a novel dimerization fold both in crystal and in solution. Moreover, we found that the dimerization of MRG chromodomains is conserved in green plants. Our findings may provide new insights into the mechanism of MRGs in regulation of gene expression in green plants.

**Electronic supplementary material:**

The online version of this article (doi:10.1007/s13238-016-0310-5) contains supplementary material, which is available to authorized users.

## Introduction

The members of human MORF4 related gene (MRG) family are highly conserved from yeast to human, including human retinoblastoma binding protein 1 (RBP-1) and the MORF4 related gene on chromosome 15 (MRG15), drosophila male specific lethal 3 (Msl3), *S. pombe* altered polarity 13 (Alp13), and *S. cerevisiae* Esa1 associated protein 3 (Eaf3p) (Bertram and Pereira-Smith, 2001). Human mortality factor 4 (MORF4) gene was identified in 1999, which can induce senescence in immortal cell lines (Bertram et al., 1999). There are seven genes with related sequences of MORF4 gene in human, which was referred as MRG family. The most studied instance in this family is MRG15 in humans, which plays crucial roles in various cellular processes such as replicative senescence, cell proliferation, and DNA damage repair, and it also exhibits functions in various complexes that regulate transcription (Leung et al., 2001; Tominaga et al., 2005; Hayakawa et al., 2010). MRG15 was identified in the two distinct nucleoprotein complexes of MAF1 and MAF2 via sucrose gradient analysis (Pardo et al., 2002). The latter is a histone acetyltransferase (HAT)-associated Tip60/NuA4 complex. Furthermore, MRG15 was also found in the Sin3S/Rpd3S complex which is a histone deacetylase (HDAC)-associated complex with antagonizing function (Yochum and Ayer, 2002). Additional studies showed that MRG15 was associated with other complexes beyond transcriptional regulation. For example, MRG15 physically interacted with PALB2 in homologous recombination of DNA repair (Hayakawa et al., 2010). Other MRG proteins have also been reported to be involved in chromatin regulating complexes. *Drosophila* Msl3 is a subunit of the male specific lethal complex (MSL) (Gorman et al., 1995). MSL has HAT activity and is required for the dosage compensation of X-linked genes in male flies (Hilfiker et al., 1997).

There are two homologues of MRG proteins in green plants, MRG1/2 in *Arabidopsis* and MRG701/702 in rice. MRG2 and MRG702 have been reported to be involved in floral transition, a highly regulated process in plant development between the vegetative stage and reproductive stage (Bu et al., 2014; Jin et al., 2015). In *Arabidopsis*, the two flowering genes of *FLOWERING LOCUS C (FLC)* and *FLOWERING LOCUS T (FT)* function antagonistically to control the flowering time (Searle et al., 2006). MRG2 directly binds to *FT* and increases the expression of *FT* in an H3K36me3-dependent manner. MRG2 also physically interacts with CONSTANS (CO), a transcription factor which functions in the regulation of photoperiodic flowering, and activates the expression of *FT* (Putterill et al., 1995; Samach et al., 2000). Moreover, MRG1/2 activates gene expression by interacting with the HAM1 and HAM2, the histone H4 specific acetyltransferases (Xu et al., 2014).

MRG family proteins contain a chromodomain at the N-terminal and an MRG domain at the C-terminal end. The MRG domains form homodimers and interact with other proteins to form functional complexes (Zhang et al., 2006b; Xie et al., 2015). As a “Royal Family” member, the N-terminal chromodomain functions as a histone reader that specifically recognizes methylated histone markers (Yap and Zhou, 2011). MRG15 mediates the recruitment of functional complexes to target genes with specific histone marks (Luco et al., 2010). Animal MRG chromodomains and fungi MRG chromodomains have been reported to bind to trimethylated histone H3 at lysine 36 (H3K36me3), such as human MRG15, *S. pombe* Eaf3, and the fly homologue Msl3 (Zhang et al., 2006a; Larschan et al., 2007; Sun et al., 2008). H3K36me3 is a histone modification usually located in the middle and at the 3’ ends of transcribed coding regions (Pokholok et al., 2005; Kolasinska-Zwierz et al.,^2009^). However, there have also been reports of MRG chromodomains in plants binding to H3K4me3. H3K4me3 is a histone modification that usually accumulates at the transcription start site (TSS) and associates with actively transcribed genes at the 5’ ends (Zhang et al., 2009). For example, the *Arabidopsis* MRG2 chromodomain (MRG2^CD^) and the rice MRG702 chromodomain (MRG702^CD^) were reported to bind to H3K4me3 *in vitro* (Bu et al., 2014; Jin et al., 2015).

In order to test the histone binding specificities of other plant MRG chromodomains, we analyzed the binding affinities of two plant MRG chromodomains to various histone peptides via isothermal titration calorimetry. Most green plants have evolved to encode two homologues of the MRG family proteins. Herein, we study the binding specificities of the chromodomains of MRG701 (the homologue of MRG702 in rice) and MRG1 (the homologue of MRG2 in *Arabidopsis*). Both the MRG701 chromodomain (MRG701^CD^) and the MRG1 chromodomain (MRG1^CD^) specifically bind to H3K36me3 and H3K4me3. We also investigated the crystal structure of MRG701^CD^.

## Results

### The chromodomain of MRG701 interacts with H3K36me3 and H3K4me3 *in vitro*

**Figure 1:**
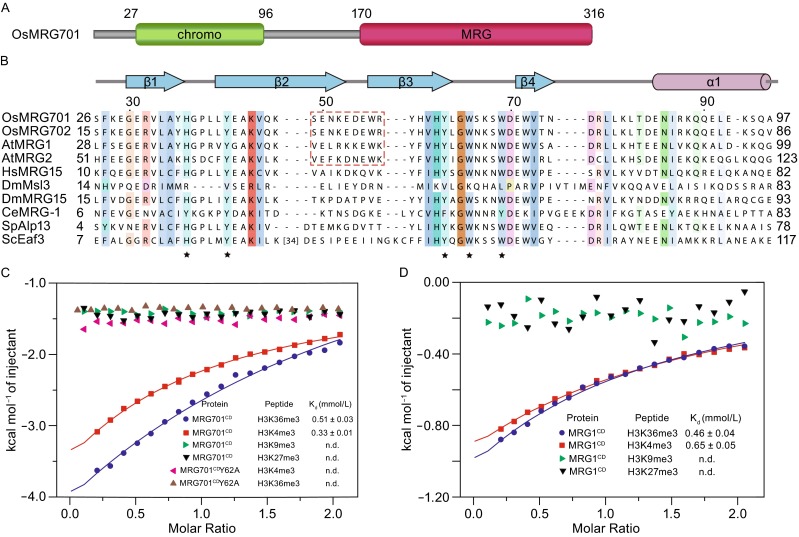
**MRG701** ^**CD**^ **specifically interacts with H3K36me3 and H3K4me3 peptides** ***in vitro***. (A) Domain architecture of MRG701 in rice. (B) Sequence alignment of the chromodomains of MRG proteins in different species. Amino acids are shaded as a function of conservation with a 30% conservation visibility cutoff that was calculated using Clustal. Secondary structural elements of MRG701^CD^ are displayed above the sequence alignment. The conserved amino acids that represent the aromatic cage for histone peptide binding are marked with asterisks. The dimerization patch is indicated with a red dashed box. A large insertion that is only present in ScEaf3 is omitted for clarity and replaced by the number of amino acids. (C) ITC measurements of binding affinities of wild-type MRG701^CD^ and mutant MRG701^CD^Y62A to tri-methylated histone peptides. (D) ITC measurements of binding affinities of MRG1^CD^ to tri-methylated histone peptides

We purified the MRG701^CD^ (residues 27–96) and MRG1^CD^ (residues 28–110) according to the domain architecture of MRG proteins (Fig. 1A). To test the binding specificities, we performed ITC to measure the equilibrium disassociation constants (K_d_). MRG701^CD^ and MRG1^CD^ both showed binding specificities to H3K36me3 (residues 31–41) as well as H3K4me3 (residues 1–9) (Fig. 1C and 1D). The measured K_d_ is summarized in Table S1, which is consistent with the previously reported values of MRG2^CD^ and MRG702^CD^ to histone peptides (Table S1) (Bu et al., 2014; Jin et al., 2015). Sequence analysis showed that there is high sequence homology between MRG chromodomains from the same species. There are only two amino acids that differ between rice MRG701^CD^ and MRG702^CD^, and 61% that differ between *Arabidopsis* MRG1^CD^ and MRG2^CD^. MRG701^CD^ shared 60% sequence identity to MRG2^CD^ and 54% to human MRG15^CD^, respectively (Fig. 1B). Five aromatic residues involved in the formation of the trimethylated lysine binding pocket are conserved in MRG701^CD^ and MRG1^CD^. These results suggest that MRG701^CD^ and MRG1^CD^ may bind histone H3K36me3 or H3K4me3 in the same manner as MRG2^CD^.

### Overall structure of MRG701 chromodomain

**Figure 2:**
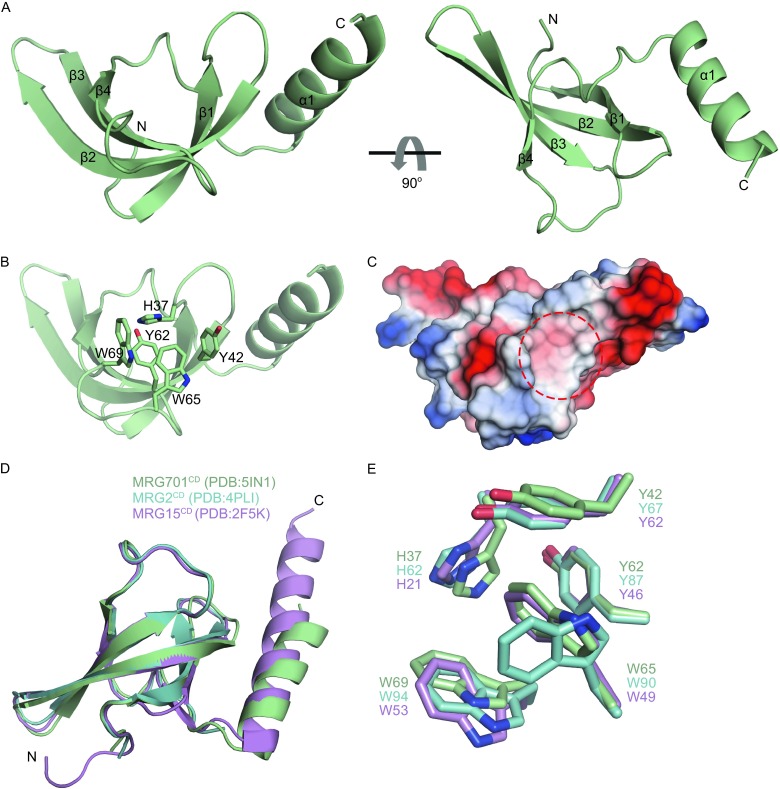
**Overall structure of MRG701** ^**CD**^. (A) MRG701^CD^ monomer consisting of four β-strands and one α-helix. View of 90° rotation around the horizontal axis is shown on right. (B) Residues consisting of aromatic cage are shown as sticks. (C) Electrostatic potential surface of MRG701^CD^ with histone binding cage indicated in red dashed circle (scale blue to red; positive to negative). (D) Structures of MRG701^CD^, MRG2^CD^, and MRG15^CD^ are superimposed and colored in light green, cyan, and violet, respectively. (E) Comparison of the aromatic cages of MRG701, MRG2, and human MRG15. Residues are shown as colored sticks as in (D)

In order to investigate the molecular mechanism of the histone peptide recognition of MRG701^CD^ and MRG1^CD^, we co-crystallized them with the histone peptides H3K36me3 (residues 31–41) and H3K4me3 (residues 1–9). Only MRG701^CD^ in complex with H3K36me3 produced crystals that diffracted to 1.4-Å. The structure was determined by molecular replacement using MRG2^CD^ (PDB: 4PLI) as a search model (Bu et al., 2014), and the structure was then refined via Phenix (Adams et al., 2010) (Table 1). There are two MRG701^CD^ molecules (molA and molB) in one asymmetric unit (AU), and the r.m.s.d. between these two molecules is 0.140 Å. The structure of MRG701^CD^ adopts a compact fold with four β-strands shaped like a β-barrel and a C-terminal α-helix (Fig. 2A). The trimethylated lysine binding pocket was formed by the following five aromatic residues: H37, Y42, Y62, W65, and W69 (Fig. 2B and 2C). The backbone Cα alignment of MRG701^CD^, MRG2^CD^, and MRG15^CD^ showed that the backbone Cα of MRG701^CD^ matches the backbone Cα of MRG2^CD^ and MRG15^CD^ quite well, with r.m.s.d. of 0.461 and 0.690 Å, respectively (Fig. 2D). The aromatic cage of MRG701^CD^ is also conserved with those of MRG2^CD^ and MRG15^CD^ (Fig. 2E). However, no extra electron density was observed in the trimethylated lysine binding pocket. Comparing the structure with other MRG chromodomain structures, we found that W65 in the aromatic cage was stretched toward the center of the cage. The indole ring of W65 occupied the binding site of the trimethyl group in the lysine side chain. Therefore, there are only MRG701^CD^ molecules in the crystal structure (Fig. S1). In the crystal structure of MRG15^CD^, the side chain orientation of the corresponding W49 is the same as that of W65 in MRG701^CD^. W65 in the aromatic cage may function as a “switch.” When there is no peptide binding, the side chain of W65 is rotated toward the center of the aromatic cage and block the binding of trimethylated histone peptides. Upon binding, the side chain of W65 is most likely flipped and leaves space for histone binding (Fig. S1).

**Table 1:** Crystallographic statistics of MRG701^CD^

Data collection	
Space group	P2_1_
Cell dimension	
a, b, c (Å)	31.79, 59.80, 40.62
α, β, γ (°)	90.00, 102.58, 90.00
Wavelength (Å)	0.9782
Resolution range (Å)^a^	29.90–1.40 (1.45–1.40)
Completeness (%)^a^	98.6 (98.8)
Rmerge (%)^a^	6.0 (10.0)
I/σI	29.0 (12.3)
Redundancy	5.2 (4.8)

Refinement	
Resolution range (Å)	29.90–1.40
No. of reflections	28,842
Rwork (%)/Rfree (%)	18.22/20.45
R.m.s.deviations	
Bond length (Å)	0.007
Bond angles (°)	1.05
Ramachandran plot	
Most favored region (%)	100
Allowed region (%)	0.0
Outliers (%)	0.0

^a^Values in parentheses are for the highest-resolution shell

### MRG701^CD^ and MRG2^CD^ showed dimerization in crystal

**Figure 3:**
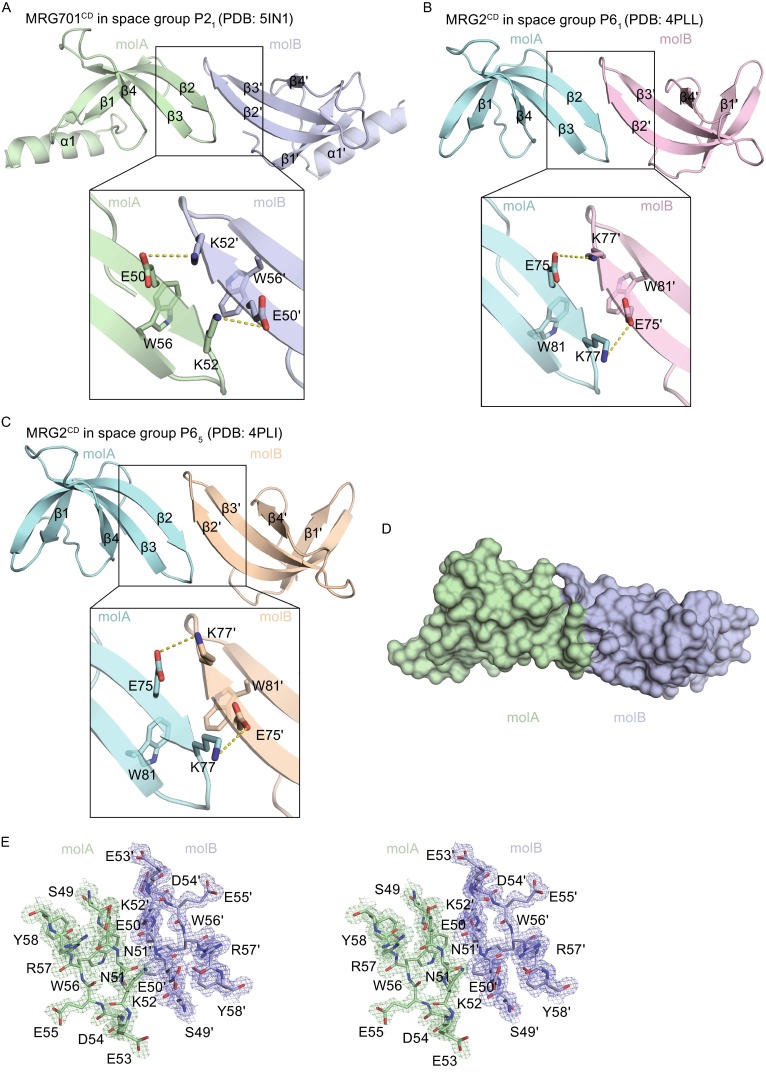
**MRG701** ^**CD**^ **and MRG2** ^**CD**^ **showed dimerization in crystal**. (A) View highlighting interactions between two molecules of the MRG701^CD^ dimer colored in light green and light blue. Yellow dashed lines indicate the hydrogen bonds. (B) MRG2^CD^ dimer in the P6_1_ space group is colored in cyan and light pink. (C) MRG2^CD^ dimer in the P6_5_ space group is colored in cyan and beige. (D) Surface structure of MRG701^CD^ dimer is shown in colored as (A). (E) The Electron density map (2FoFc) of the MRG701^CD^ dimer interface is shown in mesh at 0.5-σ and is colored as in (A)

By comparing MRG701^CD^ with other MRG chromodomains structures, we unexpectedly found dimerization in the crystal structures of plant MRG701^CD^ and MRG2^CD^ (PDB 4PLL, 4PLI, and 4PL6), but we did not find dimerization in the crystal structures of human MRG15^CD^ (PDB 2F5K) or yeast Eaf3^CD^ (PDB 3E9F) (Fig. 3A–C). In the structure of MRG701^CD^, the β2 of molA stands anti-parallel to the β2’ of molB. Specifically, both E50 and K52 on β2 of molA form both hydrogen bonding and electrostatic interactions with K52’ and E50’ on β2’ of molB, respectively. In addition, W56 on β3 of molA contacts W56’ on β3’ of molB via π-π stacking interaction (Fig. 3A and 3D). The total buried surface area of the dimeric interface in MRG701^CD^ is 645 Å^2^, which is approximately 6.9% of the total surface area of the MRG701^CD^ dimer as calculated by CNS (Fig. 3D and 3E) (Brunger et al., 1998; Brunger, 2007). MRG2^CD^ showed two different morphological crystals under the space groups of P6_1_ and P6_5_. In both kinds of crystals, the two molecules of MRG2^CD^ in one AU interact with each other in the same way as MRG701^CD^ (Fig. 3B and 3C).

### MRG701^CD^, MRG1^CD^, and MRG2^CD^ exhibited dimerization in solution

**Figure 4:**
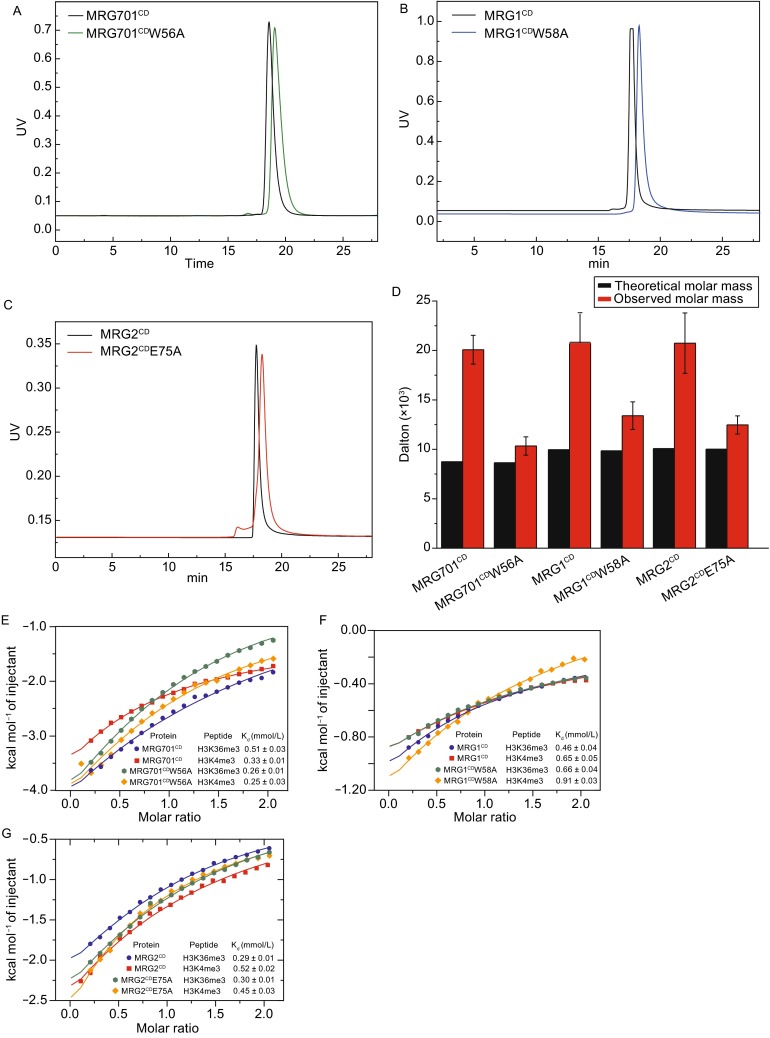
**MRG701** ^**CD**^ **, MRG1** ^**CD**^ **, and MRG2** ^**CD**^ **showed dimerization in solution**. (A–C) The elution profiles of wild-type and mutant proteins on SEC-MALS monitored via absorbance at UV 280 nm. (A) MRG701^CD^ (black) and MRG701^CD^W56A (green). (B) MRG1^CD^ (black) and MRG1^CD^W58A (blue). (C) MRG2^CD^ (black) and MRG2^CD^E75A (red). (D) The theoretical molar mass (black) and observed molar mass (red) of wild-type and mutant proteins via SEC-MALS. (E) ITC measurements for binding affinities of wild-type MRG701^CD^ and mutant MRG701^CD^W56A to tri-methylated histone peptides. (F) ITC measurements for binding affinities of wild-type MRG1^CD^ and mutant MRG1^CD^W58A to tri-methylated histone peptides. (G) ITC measurements for binding affinities of wild-type MRG2^CD^ and mutant MRG2^CD^E75A to tri-methylated histone peptides

To test whether the formation of dimerization was due to crystal packing, we designed point mutations according to the crystal structures. In each mutant, we either disrupted the π-π stacking interactions between the indole rings (MRG701^CD^W56A and MRG1^CD^W58A) or disrupted the hydrogen bonding and electrostatic interactions (MRG2^CD^E75A). The purified mutant proteins were characterized by circular dichroism spectroscopy. The resulting spectrum showed no obvious change between wild-type and mutant proteins, indicating that the overall folding of the mutants was the same as the wild-type proteins (Fig. S2). We then performed size-exclusion chromatography-coupled with the multi-angle light scattering (SEC-MALS) method to check the oligomerization status of wild-type and mutant proteins in solution. Wild-type MRG701^CD^, MRG1^CD^, and MRG2^CD^ were eluted as a single peak on the size-exclusive column with the calculated molecular weights (MW) of 20.1 kDa, 20.7 kDa and 20.7 kDa, respectively (Fig. 4A and 4B). However, the mutant proteins (MRG701^CD^W56A, MRG1^CD^W58A, and MRG2^CD^E75A) all showed an obvious delay on the size-exclusive column exhibiting MWs of 10.3 kDa, 13.4 kDa, and 12.5 kDa, respectively. The observed MWs of the mutants are nearly half the MWs of the wild-types and approximate the theoretical MWs of the corresponding monomers (Table 2). Therefore, MRG701^CD^, MRG1^CD^, and MRG2^CD^ form dimers both in crystal and in solution.

**Table 2:** Molar mass (kDa) of wild-type proteins and mutants

	Theoretical molar mass (kDa)	Observed molar mass by SEC-MALS (kDa)
MRG701^CD^	8.750	20.08 ± 1.46
MRG701^CD^W64A	8.635	10.34 ± 0.92
MRG2^CD^	10.071	20.74 ± 3.05
MRG2^CD^E75A	10.013	12.47 ± 0.92
MRG1^CD^	9.918	20.67 ± 2.99
MRG1^CD^W58A	9.803	13.36 ± 1.40

### Dimerization of the MRG chromodomains showed no effect on the binding to histone peptides

Dimerization of the MRG chromodomains through the interactions of β-strands leads to a platform with an anti-parallel histone binding site on each side of the platform. In the crystal structure, the histone binding site is embraced by the β-barrel and is spatially proximate to the dimer interface (Fig. 2B). To investigate whether the dimerization of MRG chromodomains affects the binding to histone tails, ITC experiments were performed. The measured K_d_ values of MRG701^CD^W56A to H3K36me3 and to H3K4me3 were 0.26 mmol/L and 0.25 mmol/L, respectively (Fig. 4E). These values represent less than a 2-fold change compared to those of MRG701^CD^, which were respectively 0.51 mmol/L and 0.33 mmol/L. The K_d_ of MRG2^CD^E75A and MRG1^CD^W58A to histone H3K36me3 and H3K4me3 peptides were also measured (Fig. 4F and 4G). Comparison of K_d_ showed only mild differences between the binding affinities of the wild-type and mutant proteins to histone peptides (Table S1). Thus, dimerization of the MRG chromodomains does not affect the histone binding ability.

### The dimerization of MRG chromodomains is conserved among green plants

**Figure 5:**
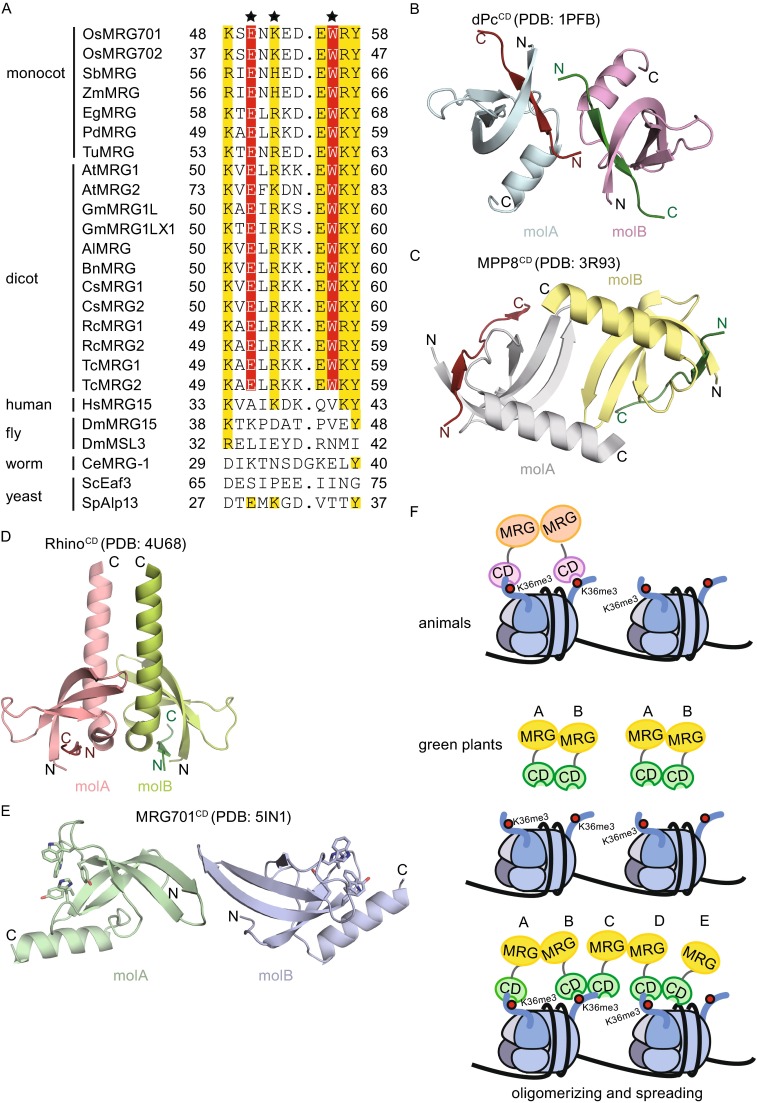
**The dimerization of MRG chromodomains is conserved among green plants**. (A) Amino acid sequence alignment of chromodomain dimerization patch between species. The conserved amino acids essential for dimer formation in plants are marked with asterisks and highlighted in colored shades by sequence identity. (B) Two molecules of the dPc^CD^ dimer colored in cyan and pink. (C) Two molecules of the MPP8^CD^ dimer colored in gray and light yellow. (D) Two molecules of the Rhino^CD^ dimer colored in light pink and light yellow. (E) Two molecules of the MRG701^CD^ dimer colored in light green and light blue. (A–C) All the bound histone H3K27me3 and H3K9me3 peptides are shown in dark orange and green, respectively. (F) Proposed model showing the different binding modes of animal and green plant MRG proteins on euchromatins

To investigate whether this MRG chromodomain dimerization may exist in a wider range of species, we performed sequence alignments via Clustal Omega (http://www.ebi.ac.uk/Tools/msa/clustalo/) using MRG701 (residues 49–59) as a reference. The alignment results indicated that residues involved in dimerization (residues E50, K52, and W56 in MRG701) are conserved in most green plants, including dicots and monocots. However, these residues were not conserved in human, fly, worm, or fungi (Fig. 5A). Therefore, this mode of dimerization of MRG chromodomains is unique to green plants.

## Discussion

In this work, we investigated the binding ability of MRG701^CD^ to the histone peptides H3K36me3 and H3K4me3, and we determined the crystal structure of MRG701^CD^. In the crystal structure we observed a noncanonical dimerization of MRG701^CD^ formed by three highly conserved residues. Mutation of the interfacial key residues showed that dimerization did not affect the binding to H3K36me3 or H3K4me3. By analyzing the structures of other chromodomains, we found that the residues involved in the formation of the dimer interface are highly conserved among green plants. These findings suggest that the dimerization of MRG chromodomains is a unique phenomenon in green plants, and this process may provide a mechanism for how MRG proteins play different roles in regulating gene expression in different species.

It has long since been established that chromodomains function as monomers, such as the human Cbx family chromodomains (Kaustov et al., 2011). Until recently, several chromodomains have been reported as a dimer. The chromodomain of *Drosophila* Polycomb (dPc^CD^) forms a dimer through the loop region connecting β4 and the C-terminal α-helix (Fig. 5B) (Min et al., 2003). The chromodomain of the M-phase phosphoprotein 8 (MPP8^CD^) dimerized through the anti-parallel β-strand paring of two molecules (Fig. 5C) (Chang et al., 2011; Li et al., 2011). Moreover, Rhino, a *Drosophila* HP1 homolog, forms a dimer via the C-terminal α-helix and the loop region between β1 and β2 (Fig. 5D) (Yu et al., 2015). Additionally, dimerization is required for the function of Rhino *in vivo*. Another example is Swi6, a *S. Pombe* HP1 homolog, which binds the H3K9me3-marked nucleosomes and drives the chromatin to switch from an auto-inhibition state to a speading-competition state (Canzio et al., 2013, 2014). In the auto-inhibited state, the chromodomain of Swi6 recognizes a histone-mimic sequence on the loop region of another Swi6 chromodomain.

Dimerization of chromodomains will juxtapose two histone binding sites and enable the chromodomains to bind two histone tails simultaneously. In the dPc^CD^ dimer, the two histone H3K27me3 peptides are proximate to each other. In the crystal structure of Mpp8^CD^, the two aromatic cages are on the exterior sides of the dimer and the two histone H3K9me3 peptides remain spatially separated. In this paper, we reported that MRG701^CD^ acts as a dimer both in crystal and in solution. Two MRG701^CD^ molecules are related via pseudo-2-fold symmetry in the crystal structure, and they form a dimer interface along the β2-strand of each molecule in an anti-parallel manner. This is a novel dimerization mode of chromodomains and the first case observed in MRG chromodomains. Dimerization of MRG701^CD^ also generates two histone binding sites in an anti-parallel way (Fig. 5E).

To date, the function of the dimerization of MRG chromodomains remains unclear. One possibility is that dimerization could regulate the recruitment of MRG proteins to their target genes. Histone H3K36me3 markers are known to locate on each side of the nucleosome and near the nucleosome core. In animals and fungi, only the C-terminal MRG domain is dimerized (MRG-MRG dimer). This leaves the N-terminal chromodomain flexible in solution and therefore accessible to the H3K36me3 markers close to the nucleosome core. However, in green plants, both the MRG domain and the chromodomain can be dimerized. If a complete dimer is formed (type AB), MRG molecules are in a rigid conformation and the two chromodomains are not easily accessible to the H3K36me3 markers. If partial dimers are formed (MRG-only or chromodomain-only), MRG molecules will oligomerize in a zig-zag manner (AB via MRG-MRG and BC via CD-CD etc.). This will result in high spatial proximity for the two methylated lysine binding pockets, and therefore the H3K36me3 markers on each side of the nucleosome would be easy to recognize and MRG molecules will spread on the chromatin surface (Fig. 5F).

The above model may explain the different function of MRG proteins between species. In mammals, yeasts, and worms, H3K36me3 marks the exons of transcribed genes and was enriched at the 3’-end of the coding region to regulate alternative splicing and transcriptional activation (Bannister et al., 2005; Pokholok et al., 2005; Kolasinska-Zwierz et al., 2009; Luco et al., 2010). In contrast, H3K36me3 is enriched at the 5’-end of the coding gene in *Arabidopsis*, and this region is also enriched in the active transcriptional marker H3K4me2/3 (Roudier et al., 2011). This suggests a different mechanism of H3K36me3 deposition and recognition (Xu et al., 2014). Our findings may aid in understanding the function of MRG proteins in green plants. Our findings show a novel mode of dimerization of MRG proteins and imply a potential diversity of the recognition mechanism of MRG proteins at the chromatin level.

## Materials and Methods

### Protein expression and purification

The cDNA encoding the chromodomain of *Arabidopsis thaliana* MRG1 (residues 28–110) and *Oryza sativa* MRG701 (residues 27–96) were amplified by PCR and cloned into the modified pET28-SMT3 vector. Point mutants were generated by Site-Directed Mutagenesis kit (NEB) according to the manufacturer’s instructions. The plasmid was transformed into *Escherichia coli* strain BL21 (DE3). The cells were cultured in LB medium with 50 μg/mL kanamycin and induced by final concentration of 0.2 mmol/L isopropyl-β-d-thiogalactopyranoside (IPTG) at 18°C for 18 h. Cells were harvested and re-suspended in 20 mmol/L Tris pH 8.0, 500 mmol/L NaCl, 25 mmol/L imidazole and then broken by cell disruptor (JNBIO). The fusion protein was purified first with Ni HiTrap column (GE healthcare) and then removed the tag by ULP enzyme. The target protein was further purified with Superdex G75 Hiload 26/60 column (GE healthcare) and then concentrated to 20 mg/mL for structural and biochemical studies.

### Crystallization and diffraction data collection

For crystallization of MRG701 chromodomain (residues 27-96) and peptide complex, a solution of 20 mg/mL protein was incubated for 1 hour with H3K36me3 (residues 31–41) in a 1:2 molar ratio. Crystallization was performed using hanging drop vapor diffusion method. Crystals of the complex were grown at 16°C by mixing 1 μL of the protein-peptide solution with 1μL precipitant solution (0.1 mol/L sodium acetate pH 4.6, 25% PEG 4000, 0.2 mol/L ammonium sulfate). The diffraction data was collected from flash-cooled crystals at 100K at SSRF (Shanghai Synchrotron Radiation Facility).

### Structure determination

The data was processed with HKL3000 (Otwinowski and Minor, 1997). The molecular-replacement solution was generated using the program Phaser and the crystal structure of MRG2 (PDB 4PLI) as a search model (McCoy et al., 2007). The initial model was built with COOT and refined using the program Phenix (Adams et al., 2010; Emsley et al.,^2010^). The final refined structure was shown by Pymol (The PyMOL Molecular Graphics System, Version 1.8 Schrödinger, LLC). Coordinate has been deposited under PDB accession code 5IN1.

### Isothermal titration calorimetry

ITC experiments were carried out at 20°C on a MicroCal iTC200. Protein and peptide were kept in an identical buffer of 20 mmol/L Tris pH 8.0, 100 mmol/L NaCl. The sample cell was filled with a 0.1–0.2-mmol/L solution of protein, and the injection syringe with 1–2 mmol/L of the titrating ligand. Each titration consisted of 20 2-μL injections with 2 min interval. Binding isotherms were analyzed by fitting data into the one-site model using the ITC data analysis module Origin 7.0 software.

### SEC-MALS analysis

The absolute molar mass of target protein was determined by size exclusion chromatography coupled with multi-angel light scattering. The experiments were performed by an Agilent system connected with a Wyatt DAWN HELEOS-II multi-angle light scattering instrument and a Wyatt OptilabrEX differential refractometer. A Wyatt 015S5 (150 Å) column was used with a 100 μL sample loop at a flow rate of 0.5 mL/min in the running buffer of 10 mmol/L Tris 8.0, 100 mmol/L NaCl. The molar mass was calculated by light scattering intensity and differential refractive index using Wyatt ASTRA 6 software (Wyatt, 1993).

### Circular dichroism spectroscopy

Circular dichroism spectroscopy was performed by Jasco J-715 spectropolarimeter. The concentration of sample was 0.2 mg/mL in 5 mmol/L Tris pH 8.0. All measurements were taken in 1mm light path length in 300 μL total volume. Spectra were collected from 180 nm to 340 nm at room temperature, with a scanning speed of 50 nm/min and corrected by the background spectra containing the same buffer.

## Electronic supplementary material

Below is the link to the electronic supplementary material.

Supplementary material 1 (PDF 385 kb)
