# Supplementary material for: Structural studies on MRG701 chromodomain reveal a novel dimerization interface of MRG proteins in green plants
Source: Protein Cell. 2016 Sep 8;7(11):792–803. doi: 10.1007/s13238-016-0310-5 (PMC5084153; doi:10.1007/s13238-016-0310-5)

Structural studies on MRG701 chromodomain reveal a novel dimerization interface of MRG proteins in green plants

Yanchao Liu, Hong Wu, Yu Yu and Ying Huang

Supplementary Tables

Table S1. K_d value (mM) of wild type proteins and mutants measured by ITC assay.

protein	peptide	K_d	△H	△S
$MRG701^{CD}$	H3K36me3	0.51±0.03	-4.8±0.19	-14.9
$MRG701^{CD}$	H3K4me3	0.33±0.01	-3.6±0.08	3.5
$MRG701^{CD}$	H3K9me3	n.d.	n.d.	n.d.
$MRG701^{CD}$	H3K27me3	n.d.	n.d.	n.d.
$MRG701^{CD}$W56A	H3K36me3	0.26±0.01	-8.2±0.12	-11.7
$MRG701^{CD}$W56A	H3K4me3	0.25±0.03	-7.1±0.33	-7.7
$MRG701^{CD}$Y62A	H3K36me3	n.d.	n.d.	n.d.
$MRG701^{CD}$Y62A	H3K4me3	n.d.	n.d.	n.d.
$MRG1^{CD}$	H3K36me3	0.46±0.04	-3.1±0.15	4.5
$MRG1^{CD}$	H3K4me3	0.65±0.05	-3.7±0.16	1.9
$MRG1^{CD}$	H3K9me3	n.d.	n.d.	n.d.
$MRG1^{CD}$	H3K27me3	n.d.	n.d.	n.d.
$MRG1^{CD}$W58A	H3K36me3	0.66±0.04	-3.7±0.15	2.0
$MRG1^{CD}$W58A	H3K4me3	0.91±0.03	-1.5±0.35	-3.8
$MRG2^{CD}$	H3K36me3	0.29±0.01	-4.0±0.05	2.35
$MRG2^{CD}$	H3K4me3	0.52±0.02	-10.4±0.25	-19.7
$MRG2^{CD}$E75A	H3K36me3	0.30±0.01	-5.6±0.11	-2.8
$MRG2^{CD}$E75A	H3K4me3	0.45±0.03	-1.9±0.06	8.9

All data were fitted into the one-site model with N value fixed 1.

Supplementary Figure Legends

Fig. S1 Aromatic cages of MRG proteins. Residues consisting of aromatic cage are shown as sticks. (A) Aromatic cage of MRG701^{CD} is shown in light green. (B) Aromatic cage of MRG15^{CD} is shown in violet. (C) Aromatic cage of MRG2^{CD} is shown in cyan with tri-methylated lysine on H3K36me3 in yellow. (D) Aromatic cage of Eaf3^{CD} is shown in orange with di-methylated lysine on H3K36me2 in yellow. (E) Comparison of the aromatic cages of MRG701, MRG2, MRG15, and Eaf3. Residues are shown as colored sticks as in (A–D).

Fig. S2 Secondary structure analysis via circular dichroism spectroscopy. Spectra of wild type protein and mutant proteins are superimposed in the UV region of 190 nm-260 nm. (A) MRG701^{CD} (black) and MRG701^{CD}W56A (green). (B) MRG1^{CD} (black) and MRG1^{CD}W58A (blue). (C) MRG2^{CD} (black) and MRG2^{CD}E75A (red).

Supplementary Figure S1

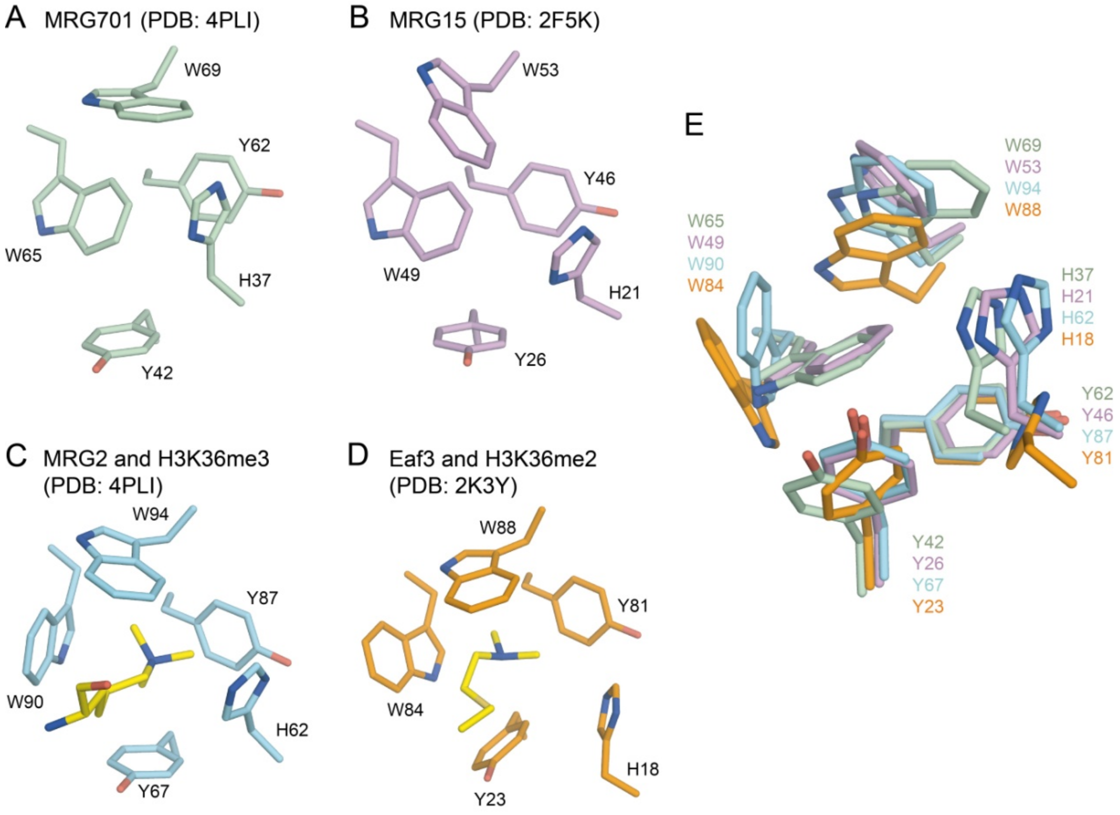

Supplementary Figure S2

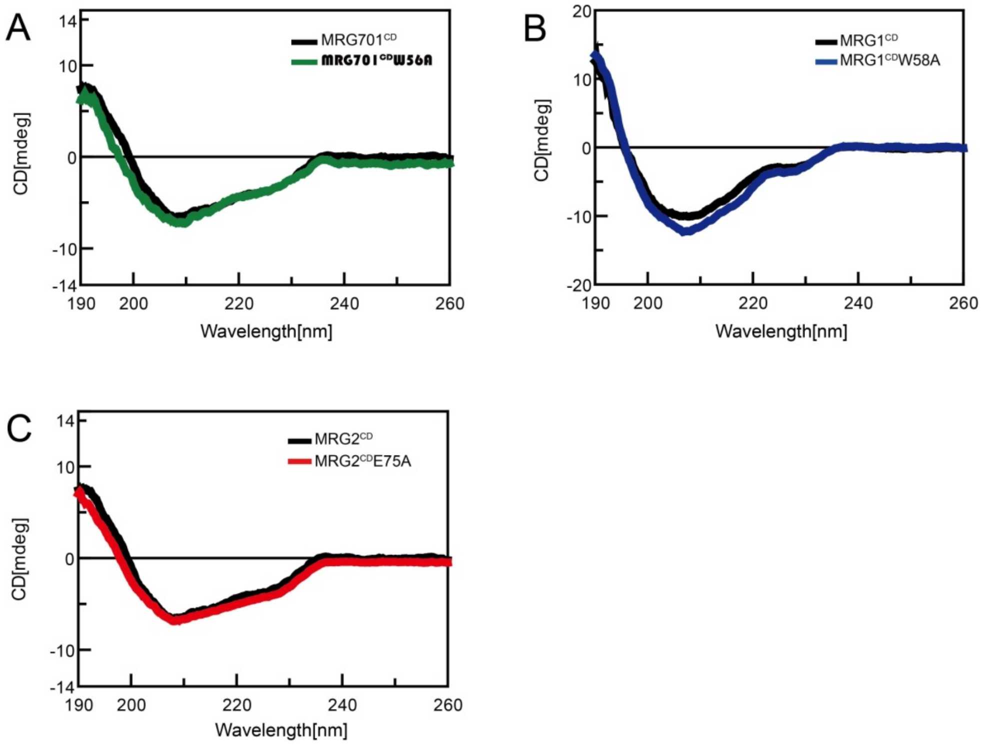

Supplement: Supplementary file 1 — Supplementary material 1 (PDF 385 kb) [file 13238_2016_310_MOESM1_ESM.pdf]
